# Sensor Networks for Aerospace Human-Machine Systems

**DOI:** 10.3390/s19163465

**Published:** 2019-08-08

**Authors:** Nichakorn Pongsakornsathien, Yixiang Lim, Alessandro Gardi, Samuel Hilton, Lars Planke, Roberto Sabatini, Trevor Kistan, Neta Ezer

**Affiliations:** 1 RMIT University—School of Engineering, Bundoora, VIC 3083, Australia; 2 THALES Australia, WTC North Wharf, Melbourne, VIC 3000, Australia; 3 Northrop Grumman Corporation, 1550 W. Nursery Rd, Linthicum Heights, MD 21090, USA

**Keywords:** human-machine system, cognitive cybernetics, cognitive states, mental workload, neurophysiology, physiological response

## Abstract

Intelligent automation and trusted autonomy are being introduced in aerospace cyber-physical systems to support diverse tasks including data processing, decision-making, information sharing and mission execution. Due to the increasing level of integration/collaboration between humans and automation in these tasks, the operational performance of closed-loop human-machine systems can be enhanced when the machine monitors the operator’s cognitive states and adapts to them in order to maximise the effectiveness of the Human-Machine Interfaces and Interactions (HMI^2^). Technological developments have led to neurophysiological observations becoming a reliable methodology to evaluate the human operator’s states using a variety of wearable and remote sensors. The adoption of sensor networks can be seen as an evolution of this approach, as there are notable advantages if these sensors collect and exchange data in real-time, while their operation is controlled remotely and synchronised. This paper discusses recent advances in sensor networks for aerospace cyber-physical systems, focusing on Cognitive HMI^2^ (CHMI^2^) implementations. The key neurophysiological measurements used in this context and their relationship with the operator’s cognitive states are discussed. Suitable data analysis techniques based on machine learning and statistical inference are also presented, as these techniques allow processing both neurophysiological and operational data to obtain accurate cognitive state estimations. Lastly, to support the development of sensor networks for CHMI^2^ applications, the paper addresses the performance characterisation of various state-of-the-art sensors and the propagation of measurement uncertainties through a machine learning-based inference engine. Results show that a proper sensor selection and integration can support the implementation of effective human-machine systems for various challenging aerospace applications, including Air Traffic Management (ATM), commercial airliner Single-Pilot Operations (SIPO), one-to-many Unmanned Aircraft Systems (UAS), and space operations management.

## 1. Introduction

**Figure 1:**
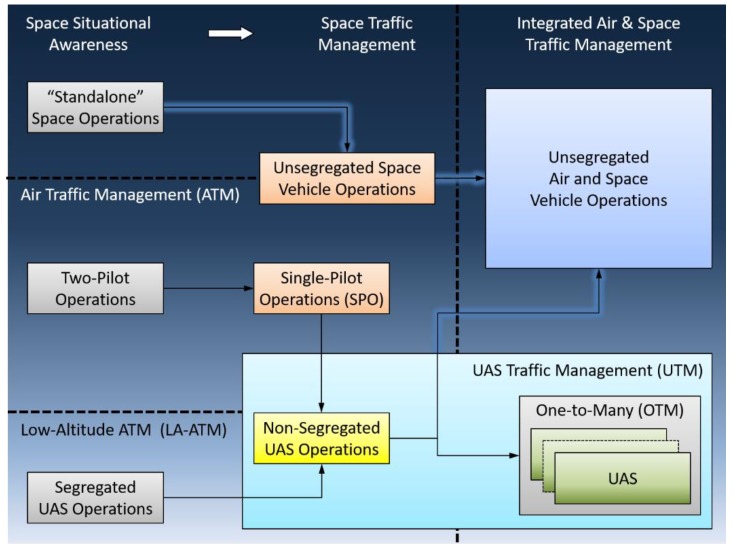
Evolution and progressive integration of conventional and autonomous air and space platforms in a cohesive UAS, Air and Space Traffic Management (UTM/ATM/STM).

Advances in aerospace Cyber-Physical Systems (CPS) are supporting a progressive evolution of conventional platforms to feature higher levels of automation and information sharing. Major benefits of these two capabilities include a progressive de-crewing of flight decks and ground control centers, as well as the safe and efficient operations of very diverse platforms in a shared, unsegregated environment. Important efforts are, for instance, addressing the integration of Unmanned Aircraft Systems (UAS) in all classes of airspace, eliciting the introduction of a UAS Traffic Management (UTM) service which seamlessly integrates within the Air Traffic Management (ATM) framework [1], especially in lower airspace. Similarly, the operation of space launch and re-entry platforms currently requires considerable airspace segregation provisions, which if continued will become increasingly disruptive to civil air traffic. Moreover, the currently limited space situational awareness is posing significant challenges to the safety and sustainability of spaceflight due to the rapidly growing amount of resident space objects and particularly orbital debris. The deployment of network-centric Communication, Navigation, Surveillance and Avionics (CNS+A) systems and their functional integration with ground-based ATM in a Space Traffic Management (STM) framework will support a much more flexible and efficient use of the airspace with higher levels of safety [2]. In terms ofair traffic, advanced CNS+A systems will support the transition from the two-pilot flight crews to a single pilot in commercial transport aircraft, with the co-pilot potentially replaced by a remote pilot on the ground. A single remote pilot on the ground, on the other hand, will no longer be restricted to controlling a single UAS and instead will be allowed to control multiple vehicles, following the so-called One-to-Many (OTM) approach [3]. Figure 1 schematically illustrates these important evolution paths.

**Figure 2:**
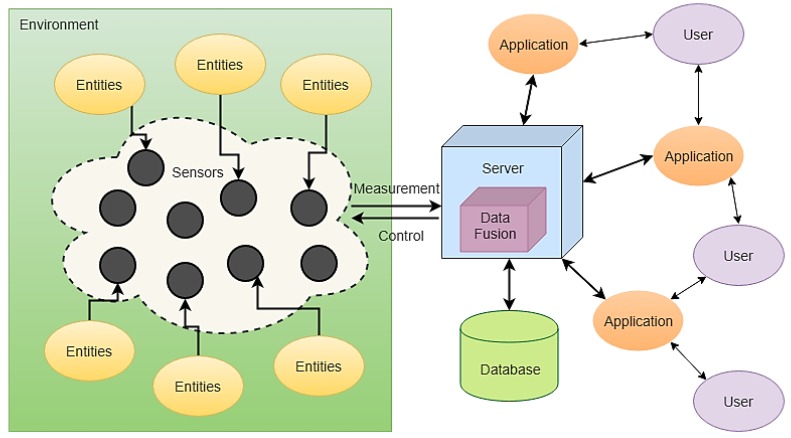
Fundamental elements of a sensor network.

Increases in automation complexity and in the amount of handled information are eliciting a need for further research in Human-Machine Interfaces and Interactions (HMI^2^) for better human-machine teaming to improve the overall system performance [4]. Important research and development efforts are focusing on monitoring and supporting the appropriate cognitive workload of human operators in complex and time-critical tasks through real-time measurement of neurophysiological variables [5,6]. In doing so, the adoption of sensor networks is both a natural and necessary evolution to effectively exchange, synchronise and process measurement data within a customisable operational network architecture. As conceptually illustrated in Figure 2, a sensor network implements three fundamental components: a *control element* to effectively regulate its functioning and particularly to ensure successful monitoring and recording of data from the environment through a suite of disparate sensors, a *computation element* to process and fuse collected data and thus generate the desired information; a *communication element* networking all sensors, databases and end users to the server to collect raw measurement data and disseminate the processed information.

**Figure 3:**
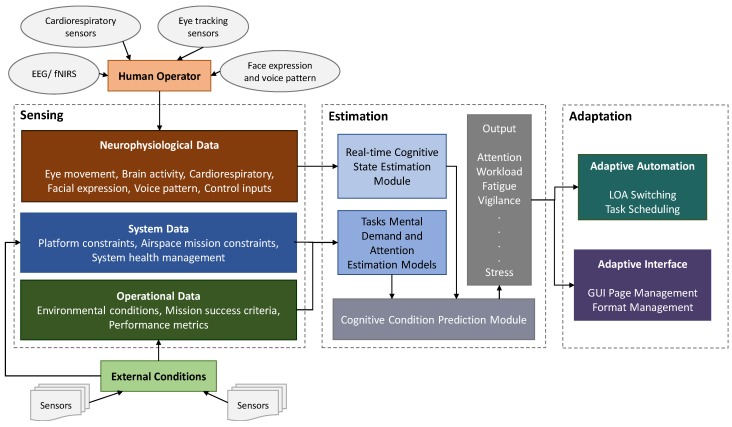
CHMI^2^ framework.

Collectively, these three essential elements (control, computation and communication) form the definition of a Cyber-Physical System (CPS). CPS are engineered systems built from, and dependent upon, the seamless integration of computational algorithms, physical components, and at the highest level, the integration of the human-machine feedback. Practical CPS combine sensors and embedded computing to monitor and control physical processes, with feedback loops that allow these processes to affect computations and vice-versa. The Cognitive HMI^2^ (CHMI^2^) concept depicted in Figure 3 provides a notable example of an advanced CPS by implementing system automation support modulated as a function of cognitive states of both the human operator as well as relevant operational/environmental observables. Initially described in [5,6], the foundation of the CHMI^2^ framework is the real-time neurophysiological sensing of the human operator to infer cognitive states and in turn drive system adaptation. This requires the adoption of three fundamental modules: sensing, estimation and adaptation. Various advanced wearable and remote sensors are exploited in the CHMI^2^ sensing module to track operators’ neurophysiological parameters in real time. The collected data is then passed to the estimation module to be processed to infer the operators’ cognitive states. Prior to operational use, the estimated cognitive states are validated in the initial calibration phase by correlating these cognitive states with objective measures of the designed scenario, such as mission performance and task complexity. Lastly, the inferred cognitive states are used by the CHMI^2^ adaptation module to dynamically adapt the HMI^2^ and automation behaviour.

One important consideration when designing CHMI^2^ and similar systems is that each neurophysiological parameter is sensitive to different biological processes and circumstances, and is affected by very different disturbances. For instance, heart rate variability is sensitively influenced by time of day, whereas blink rate and pupillometry are sensitive to ambient light stimuli. Due to the complex nature of neurophysiological phenomena, the monitoring of multiple parameters is required to accurately and reliably estimate the cognitive workload or other states of the human operator [7]. Moreover, there are additional difficulties associated when using multiple sensors: notably, each sensor has different measurement performance (e.g., accuracy, resolution etc.) and sampling frequencies. Hence, a well-designed sensor network optimisation scheme is key when designing reliable human-machine systems, not only to ensure optimal use of multiple sensors within the sensing module but also to devise a data fusion approach for increased overall inference accuracy of the estimation module.

The rest of the article is structured as follows: Section 2 presents the cyber-physical sensor networks in the CHMI^2^ framework. Section 3 describes the main neurophysiological measurements and sensors in detail, together with their performance parameters and relevance for aerospace human-machine systems. In particular, eye tracking sensors are discussed in Section 3.1, and cardiorespiratory, central nervous sensors, face and voice recognition in Section 3.2, Section 3.3, Section 3.4 and Section 3.5 respectively. Machine learning methods used in cognitive state estimation are discussed in Section 4. Section 5 details the methodology to experimentally characterise the performance of selected sensors and the propagation of measurement uncertainty through machine-learning inference systems. Section 6 describes the use of neurophysiological sensors in various aerospace applications with a focus on contemporary ones. Lastly, conclusions are drawn in Section 7.

## 2. Sensor Networks in CHMI^2^ Framework

**Figure 4:**
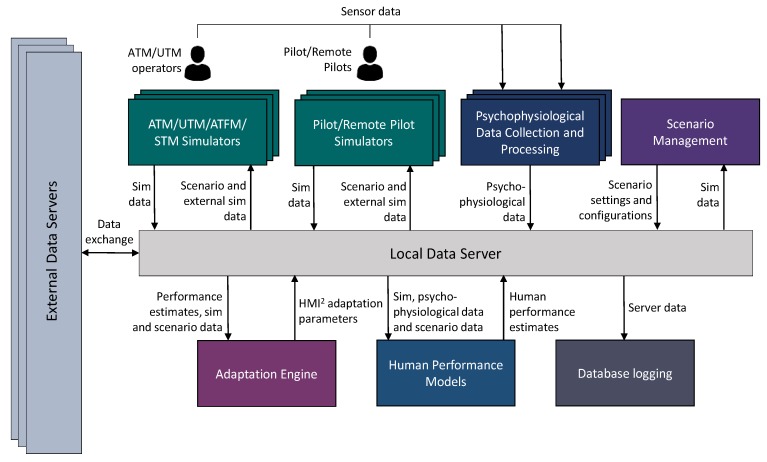
HFE Lab architecture [4].

A core component of the CHMI^2^ sensor network introduced in Section 1 is the *Human Factor Engineering (HFE) Lab* software at RMIT University, which supports the networking and data management for all the CHMI^2^ sensors and data streams. The *HFE Lab* software supports several aerospace cyber-physical system applications: ATM, Air Traffic Flow Management (ATFM), UTM, pilot/remote pilot stations, spacecraft operations control centers and STM applications. As illustrated in Figure 4, the *HFE Lab* software also caters for complex scenarios to be simulated and allows for multiple participants. Neurophysiological data together with operational data (simulated mission and scenario information) are collected and analysed offline to improve the accuracy and reliability of cognitive state estimation models.

**Figure 5:**
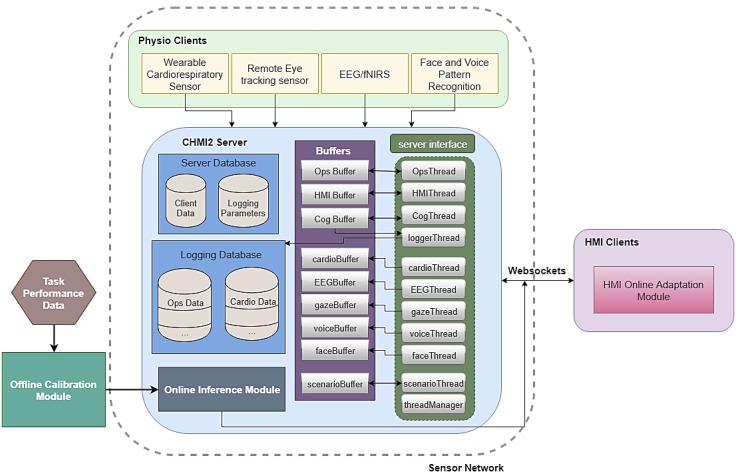
Fundamental role and components of the CHMI^2^ server as part of the HFE Lab [8].

A sensor network is effectively realised by the *HFE Lab* software that fuses neurophysiological sensor data and other environment/mission data. The information flows and data server components are detailed in Figure 5.

The neurophysiological sensors firstly obtain various physiological measurements which consist of cardiorespiratory, eye, brain, face and voice features. These sensors interact with dedicated *physio clients* which perform pre-processing functions such as data filtering and feature extraction prior to sending the data to the CHMI^2^ server. The CHMI^2^ server is the sensor network’s central element of data storage and distribution. This server synchronises incoming data from the various physio clients. Apart from neurophysiological measurements, scenario and mission data are also logged by the server. The server threads parse the data into separate buffers that are read by other threads, which are then logged into different databases. The threads include recurrent data management function as *loggerThread* and *threadManager*. In order to run different functions simultaneously in the server, a suitable thread management facilitates effective communication with the *HMI clients*. Furthermore, the different aerospace simulators present in Figure 4 may not always allow all neurophysiological sensors in *HFE Lab* to be exploited. For example, the lab’s remote eye tracking sensor is limited to use on desktop PCs and is not applicable in the 210° flight simulator. Hence, the sensor network architecture of *HFE Lab* provides substantial flexibility in the integration of different types of neurophysiological sensors as the software is modular and modifications to individual sensor threads can be made to cater for custom sensor data.

## 3. Neurophysiological Sensors

This section describes in detail the state-of-the-art in neurophysiological sensing technologies that are most commonly used in aerospace applications, including: eye-tracking, cardiorespiratory and central nervous system monitoring devices. The key neurophysiological measurements used in this context and their relationship with the operator’s cognitive states are discussed. Emotional state estimation based on face expression and voice pattern analysis are also discussed.

### 3.1. Eye Tracking Sensors

**Figure 6:**
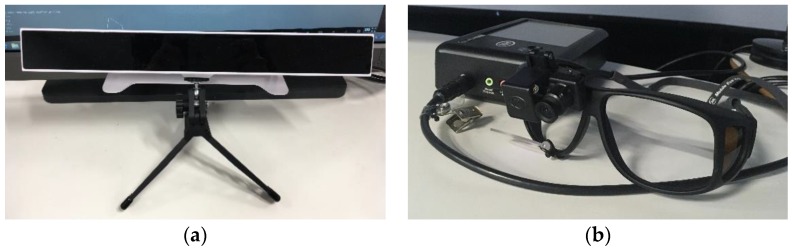
Eye tracking technologies used in *HFE Lab*. (**a**) remote sensor, (**b**) wearable sensor.

Eye tracking is capable of providing both passive and active control, supporting closed-loop human-machine interactions. Passive control supports adaptive HMI^2^ formats and functions by assessing the behaviour and functional state of the operator through software running in the background, whereas active control allows human operators to interact directly with the machine by providing gaze-based control inputs. While passive control requires eye tracking data to be further processed and fed into an inference engine to determine the operator’s functional state, active control directly uses the raw data and is therefore more straightforward in terms of system implementation. A number of issues affect both types of control and are associated to the performance of eye tracking systems. These issues should be carefully considered before utilising eye tracking technology in aerospace applications. For instance, these issues could include the inadvertent activation of gaze-based control, as well as low reliability, accuracy and repeatability of eye tracking measurements. There are two types of eye tracking technologies; wearable and remote sensors (Figure 6).

Wearable sensors are not limited by a Field of View (FOV) and this characteristic is very advantageous for eye tracking in open environments, such as flight decks, where the user may be looking at displays as well as physical controls and out of the window. On the other hand, the use of remote sensors might require limiting the movement of the operator’s head and/or gaze, and while this can result in increased accuracy, it is typically operationally restrictive. Additionally, some remote sensors can also detect the distance between the operator and the screen, which can be useful for HMI design optimisation and neuro-ergonomics studies. In a recent work, we targeted the full performance characterisation of both types of sensors. The study showed that the *HFE Lab’s* remote sensor performs better in term of accuracy and precision [9]. The findings of this study are reviewed and discussed in Section 4.1. We also note that remote sensors may enjoy greater operator acceptance in deployed systems.

Eye tracking features are sub-divided into gaze features and pupillometry. Gaze features comprise of fixation, saccade, dwell, transition and scan path. Fixation is a gaze state that fixed or focussed on an object *(x, y)* at time *(*$t_{fn}$*).* The rapid and small eye movements between fixations are saccades ($s_{n}$) which can be defined by saccade velocity ($v_{s_{n}}$) and saccade time ($t_{s_{n}}$). In the domain of pupillometry, the three most important features are eye closure, blink rate and pupil radius. With respect to gaze features, the gaze position allows to derive additional features such as fixation and saccades, from which more complex features can be extracted, such as visual entropy. The visual entropy (H) can be determined from gaze transitions between different Region-of-interest (ROI), which are typically represented by a matrix. For instance, $p\left(Y_{ij}|X_{i}\right)$ is the probability of a transition between *ROI_i_* to *ROI_j_* given a previous fixation on *ROI_i_* and $p\left(X_{i}\right)$ is the probability of a fixation being on *ROI_i_* [10]. The Nearest Neighbour Index (NNI), quantifies the randomness based on fixations, while the explore/exploit ratio [11] computes the randomness based on a combination of saccades, long and short fixations. For a given fixation distribution, the NNI is given by the ratio of the mean nearest neighbour distances (${\bar{r}}_{A}$) and the mean random distances (${\bar{r}}_{E}$) [12]. Table 1 summarises the parameters used to evaluate eye activity [10,13,14,15,16].

**Figure 7:**
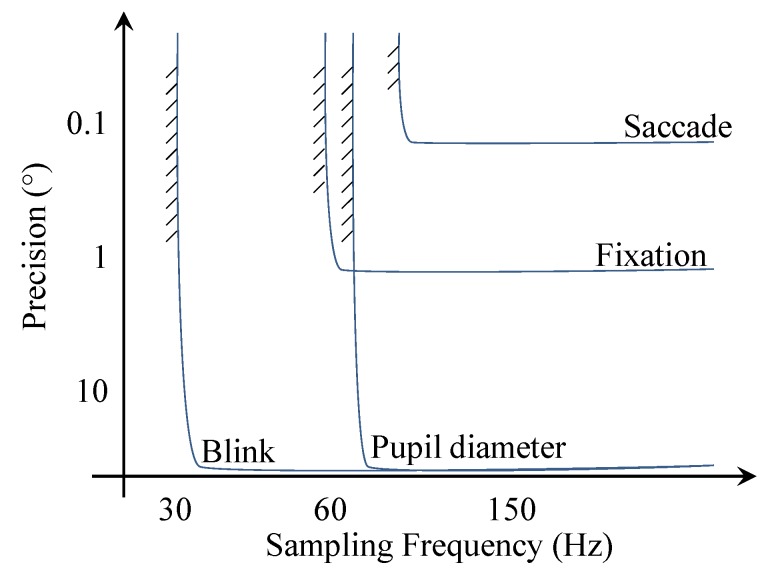
Detectability of main eye activity features as a function of sensor precision and sampling frequency.

Sampling frequency, accuracy, latency and precision are the most important properties for characterising eye tracking systems [22]. As illustrated in Figure 7, the minimum sensor performance requirements are different for each measured parameter. Saccade is the most difficult feature to measure since it requires the sensor to have both high sampling frequency and high precision, whereas blink rate can be captured even at lower frequencies and at very low precision.

Passive control methods can exploit various eye activity variables such as fixations, blink rate, saccades, pupil diameter, visual entropy and dwell time, which are related to the operator’s cognitive state [22,23] as shown in Table 2. Arrows represent the changes of the variables when there is an augmentation of the cognitive states; an increase (↑) or decrease (↓), and dashes (-) present an uncertain/negligible effect.

### 3.2. Cardiorespiratory Sensors

**Figure 8:**
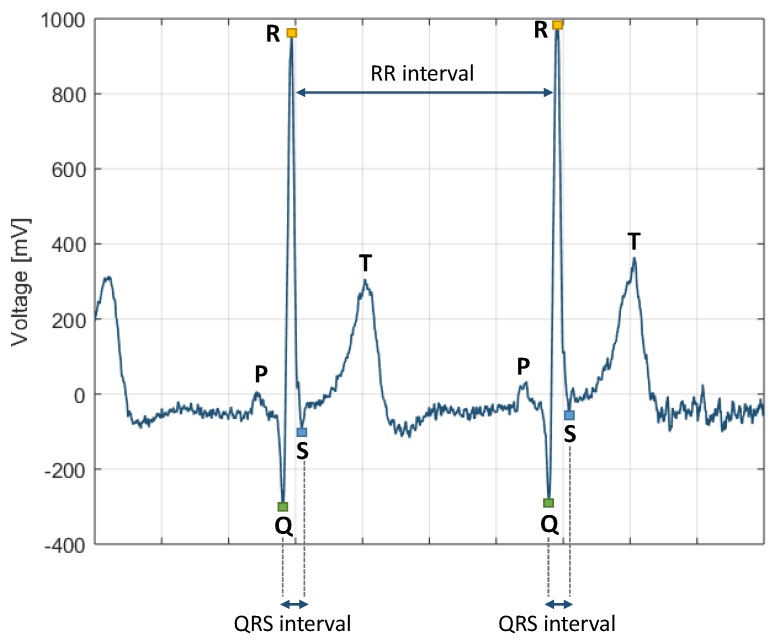
QRS complex configurations.

A cardiorespiratory sensor is a biological telemetering system which can be operated in either real-time data transmitting or data logging mode. The usage of the sensor is primarily to monitor heart and respiratory activity. The cardiac monitoring is typically based on electrocardiography (ECG), which exploits electrodes in contact with the skin. Heart muscle depolarisations and polarisations generates electrical waves that propagates towards the skin and can be measured by the electrodes. Other cardiac monitoring techniques include hemodynamic sensors, which look at blood flow characteristics such as pressure and flow rate. Conventional ECG-based cardiac sensors use electrode pads, which have to be applied carefully and may be detached by sweat, while the latest wearable sensors are based on conductive fabric to measure ECG. The heart impulses are represented by waves of P-QRS-T deflection as illustrated in Figure 8. Atrial depolarisation is represented by the P wave while the QRS wave complex obscures atrial repolarisation. Ventricular repolarisation is represented by the T wave and ventricular depolarisation is represented by QRS wave complex [24]. The R wave is the largest wave which allows to accurately extrapolate the time interval (in seconds) between two consecutive heart beats, hence called R-to-R (RR) interval.

The *HR* is given in beats per second as:

(14) $$HR=\frac{60}{RR\;interval}$$

Another important cardiac activity metric is the HRV which tracks variations between two consecutive beats. HRV can be analysed in time, frequency and geometric domains. Time-domain measures quantify the variability in the interbeat interval (IBI), given in milliseconds (ms), which is the time period between successive heartbeats. IBI is similar to RR and *Normal-to-Normal* (NN). The difference between RR and NN is that NN refers to the RR interval of normal beats only, with the abnormal beats removed. Table 3 details the various HRV metrics in time-domain measurements with associated equations.

Frequency domain metrics are extracted through spectral analysis of the RR interval data to obtain the power spectrum density (PSD) estimate of a given time series. The PSD is divided into four frequency bands [26] as shown in Table 4: the Ultra-Low Frequency (ULF), the Very-Low Frequency (VLF), the Low Frequency (LF) and the High Frequency (HF). In particular, the HF and LF bands occur due to the heart’s control of the sympathetic and parasympathetic branches of the autonomic nervous system. The HF band represents the activity of the sympathetic branch, which regulates the relaxation (‘rest and digest’) functions of the body, while the Low Frequency (LF) component represents the activity of the parasympathetic branch, which regulates the action (‘fight or flight’) functions in the body. During activity (both physical and mental), the power in the LF band has been observed to increase in proportion to the HF band, with the LF/HF ratio being an important indicator of the relative powers between the two bands. VLF is sometimes used for recordings over five minutes, when considering longer-term, ULF is also added in certain calculations.

Geometric metrics analyse the HRV by converting RR intervals into geometric plots. Poincaré plots display the correlation between consecutive RR intervals, with *RR*(*i*) plotted on the *x*-axis and *RR*(*i*+1) plotted on the *y*-axis. The points are distributed in an elliptical manner along the plot with *SD*1 and *SD*2 respectively defined as the minor and major axes of the ellipse. *SD*1 reflects the short-term characteristics of HRV (i.e., the variability over successive beats) while *SD*2 reflects the long-term characteristics of HRV (i.e., the variability over multiple beats). *SD*1 and *SD*2 are given by [27]:

(26) $$SD1=\sqrt{0.5\cdot Var_{n}\left(RR_{i}-RR_{i+1}\right)}$$

(27) $$SD2=\sqrt{0.5\cdot Var_{n}\left(RR_{i}+RR_{i+1}\right)}$$

where *n* is the sample window and is usually set to 30 s. The *x* and *y* coordinates of the ellipse are given by the parametric equation:

(28) $$\left[\begin{array}{c} x\\ y \end{array}\right]=\sqrt{2}\cdot \left[\begin{array}{cc} \cos\left(\pi /4\right) & -\sin\left(\pi /4\right)\\ \sin\left(\pi /4\right) & \cos\left(\pi /4\right) \end{array}\right]\cdot \left[\begin{array}{c} SD2\cdot \cos\left(\theta \right)\\ SD1\cdot \sin\left(\theta \right) \end{array}\right]+\left[\begin{array}{c} {\bar{RR}}_{i}\\ {\bar{RR}}_{i+1} \end{array}\right],\;0<\theta <2\pi$$

**Figure 9:**
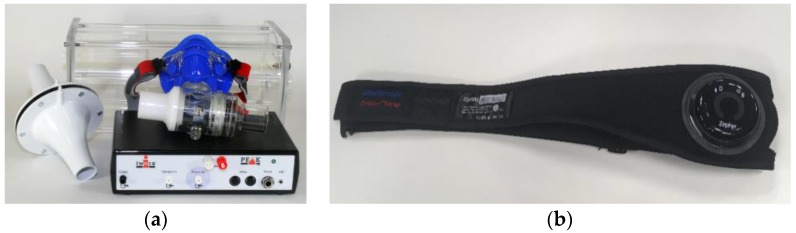
Respiratory technologies. (**a**) airflow, (**b**) strain gauge.

Concerning respiratory activity monitoring, there are two main types of devices: strain gauges and airflow sensors, illustrated in Figure 9. The most common one is strain gauges because this system is less expensive, unobtrusive and easier to use. The mechanical strain from the strap is converted into voltage. An airflow technique requires participant to wear a mask or tube while breathing. It measures the oxygen consumption and carbon dioxide production.

**Figure 10:**
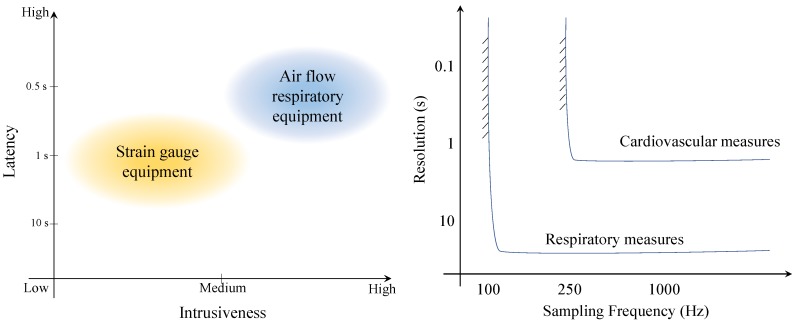
**Left**: performance comparison of the different respiratory monitoring technologies. **Right**: detectability of cardiorespiratory features as a function of sensor resolution and sampling frequency.

As illustrated in Figure 10 (Left), the advantage of strain gauge equipment is that it has low intrusiveness than the airflow. However, airflow-based devices have better latency. Moreover, the minimum sensor performance requirements are different for each parameter. The main performance factors to be considered include resolution and sampling frequency. Compared to cardiovascular measures, respiratory measures require lower sampling frequency since cardiovascular parameters such as HRV require millisecond resolution. Due to their lower intrusiveness, strain gauge wearable sensors are mainly discussed here.

The most common respiratory variable is BR, also referred to as the breathing or respiratory rate, which is typically expressed in breaths per minute. Other variables include the respiratory amplitude, expressed in terms of Tidal Volume (TV) and Minute Ventilation (MV). These three variables are detailed in Table 5.

A number of studies focused on the characterisation of cardiorespiratory sensor performance [28,29,30,31,32]. Some of the key results of an experimental characterisation activity targeting both physical and mental workload are presented in Section 4.2. Several studies revealed that heart and respiratory parameters are reliable measures of the operator’s cognitive states. Table 6 summarises the various cardiorespiratory variables that used to estimate cognitive states [3,33,34,35,36]. Arrows represent the changes of the variables when there is an augmentation of the cognitive states; an increase (↑) or decrease (↓), and dashes (-) present an uncertain/negligible effect. For instance, when the level of Mental Workload (MWL) increases, HR increases (↑). However, some parameters were found to be moderated by training and experience. For instance, compared to the baseline, a substantial suppression in the HF band in a medium task load condition could be observed. However, an ATC task of equivalent difficulty that was undertaken by inexperienced and experienced participants demonstrated that the HR of inexperienced participants was not noticeably affected by changes in level of complexity in ATC tasks [37], and this might stem from lack of attention and understanding in situational awareness.

Additionally, the disadvantage of using cardiorespiratory variables is their slow response to the rapid changes in cognitive states compares to other variables [38].

### 3.3. Neuroimaging Sensors

**Figure 11:**
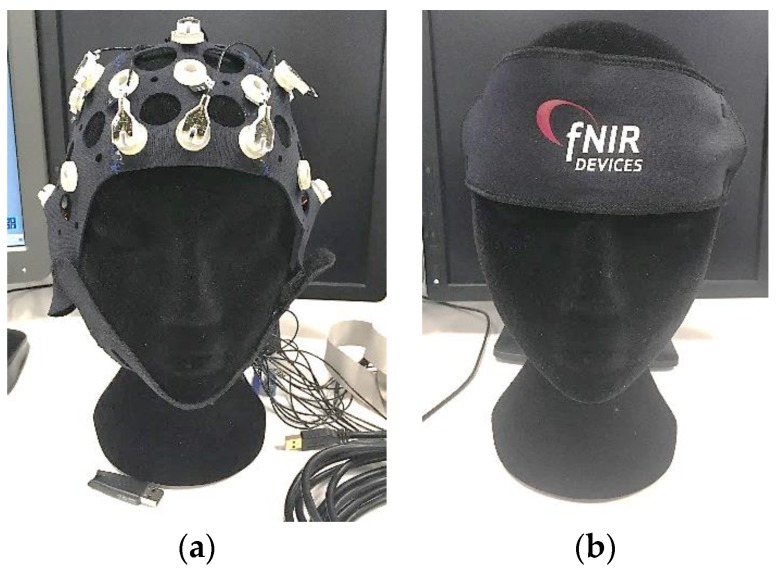
Medical-/research-grade neuroimaging systems: (**a**) EEG; (**b**) fNIRS.

Neuroimaging technologies are used to monitor and better understand the brain workings. The recent technological developments in this domain are opening new avenues for aerospace human factors engineering research and development. The increasing commercial availability of mobile/wearable brain sensing devices (Figure 11) has resulted in many opportunities for neuroergonomic studies. This paper however only focusses on two techniques [39]: EEG including its spectral analysis and Functional near-infrared spectroscopy (fNIRS).

These techniques can be divided into two main categories for achieving neuroimaging, namely the direct observation of neural activity in a response to stimuli, and the indirect metabolic indicators of neural activity [39]. The former technique includes sensors such as EEG that record the electrical activity in the brain generated by firing neurons [40]. The latter technique includes sensors such as fNIR which uses a spectroscopic method to determine levels of blood oxygenation in the cortex of the brain [41]. Neuroergonomics differs from traditional neuroscience in the way that it investigates the brains function in response to work. Hence, the neuroergonomic method implemented is required to be flexible so that it can adapt to the specific application [39]. Table 7 details further categorisations of the two techniques based on their temporal and spatial resolution.

Table 8 summarises brain-related estimation techniques related to cognitive states. Conventional EEG techniques utilise spectral analysis, decomposing the raw signal into different frequency bands and comparing the relative strength between different bands. For instance, attention can be determined when the beta spectrum is high and alpha spectrum is low in the pre-frontal (Fp1) position [49,50]. More advanced techniques such as regression and neural networks were later introduced for large data analysis.

**Figure 12:**
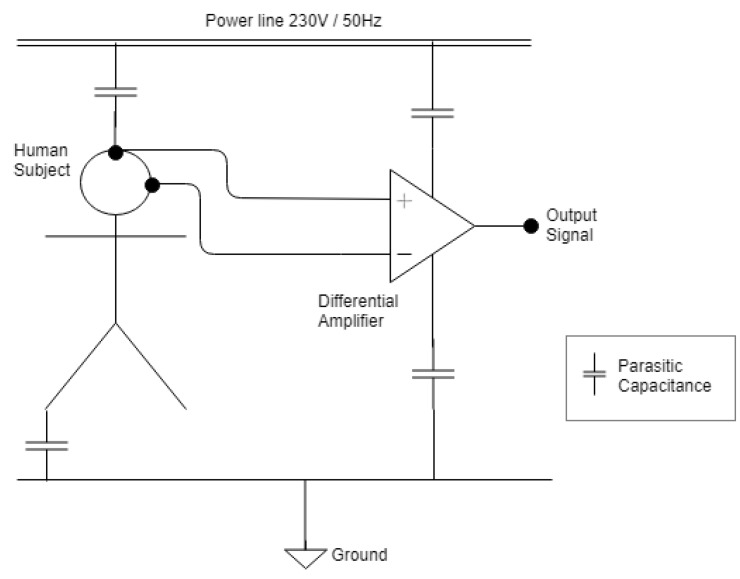
EMI induced by mains power. Adapted from [102].

Additional challenges associated with the EEG specifically includes the artifacts and Electromagnetic Interference (EMI). The EEG signals of interest have a frequency ranging from 0.01 to approximately 100 Hz with a voltage changing from a few microvolts to around 100 µV [101]. As the amplitude is very small, the EEG signal is especially prone to EMI. The noise attributed to the EEG signal can come from a variety of different artifacts and can either be electromagnetic noise caused by neurophysiological or non-neurophysiological sources [102]. The neurophysiological artifacts most frequently originate from ocular, muscular or cardiac contaminants [101]. Non-neurophysiological sources stem from external artifacts that are prominently caused by power line interference, this can be observed at 50/60 Hz in the spectral analysis [102,103]. As schematically illustrated by the electric circuit diagram in Figure 12 the result of this is a parasitic capacitance between the power line and subject/measurement equipment. The EMI thus interacts with the human body and the measurement cables which function as an antenna for the electromagnetic contamination [103]. Some of the more prominent causes for power line interference stems from fluorescent lamps 1–2 m away from the EEG device [102,104]. Additional non-neurophysiological artifacts include instrumentation artifacts. These are artifacts that stem from within the electronics, and are observed as thermal noise, shot noise or 1/f (pink) noise [103]. As the EEG equipment is highly susceptible to artifacts, proper procedures to prevent these effects need to be considered to obtain accurate EEG recordings.

### 3.4. Voice Patterns

**Figure 13:**
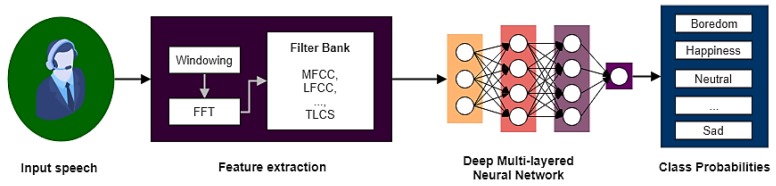
Top level architecture of speech analysis systems based on pitch and energy. Adapted from [105].

In most aerospace applications, voice communications have a key role in various operational tasks. It is therefore desirable to implement speech recognition and pattern analysis due to its various advantages; chiefly the fact that specific equipment is not required and it is an inherently unobtrusive process. Early studies mainly focused on speech emotion recognition [105,106,107,108,109]. A prototypical speech analysis system based on pitch and energy is presented in Figure 13. Other prosodic and linguistic features can also be exploited for voice pattern analysis.

The most common speech analysis methodology is to firstly convert input sound signals into power spectrum by different filter banks such as Mel-Frequency Cepstrum Coefficients (MFCCs), log-frequency power coefficients (LFPCs) and Two-Layered Cascaded Subband Cepstral Coefficients (TLCS). The MFCCs exploits Fast Fourier Transform (FFT) to get a power spectrum, which then maps the power to the mel-scale. Hence, the MFCCs represents the amplitude of the spectrum by taking discrete cosine transform to the log power of mel frequencies [110]. LFPCs are similar to MFCCs but consider all frequency ranges equally unlike MFCCs [111]. By comparing three of these filters, TLCS outperform MFCCs and LFPC because it cover wider ranges of frequency and consider both inter-subband and intra-subband energy [112]. The largest emotion diversity occurs at 0–250 Hz in low frequency and 2.5 kHz–4 kHz in high frequency [113]. Moreover, the spectrogram representation can also assist in determining the range of frequencies representing in red high amplitude, in green mid amplitude and in blue low amplitude signals [113]. After completing feature extraction, a machine learning algorithm is employed to infer emotions from spectrograms. Deep neural networks have been used rather successfully for this particular process. The most common architectures are recurrent neural networks and feed-forward neural networks. For feed-forward architectures, the Convolution Neural Network (CNN) is increasingly used [105].

However, emotional states are potentially related to cognitive states and a recent study presented the estimated cognitive load from voice pattern [114], though the methodology used was different from emotion recognition, and involved a Support-Vector Machine (SVM). SVM is a machine learning algorithm which requires labelled trained data. In order to define cognitive load, self-assessed workload was involved in the scenario to label the training data. The participant can rate the level of workload in real time. This can determine when the operator is overloaded which is a very important feature in human-machine systems.

### 3.5. Face Expressions

Face expression analysis is another common method to evaluate human emotional states. Similarly to voice patterns, face expression analysis does not require specialised equipment beyond an optical camera (e.g., RGB) and it is unobtrusive. Face expression mainly relies on a deep neural network, and the most popular model is the CNN. In such approach, the input image is convolved in the convolution layers to generate a feature map through a filter collection. Fully connected networks are then combined into feature maps. The last layer before the output layer is *softmax* algorithm which recognises face expressions by their class-based layers [115].

**Figure 14:**
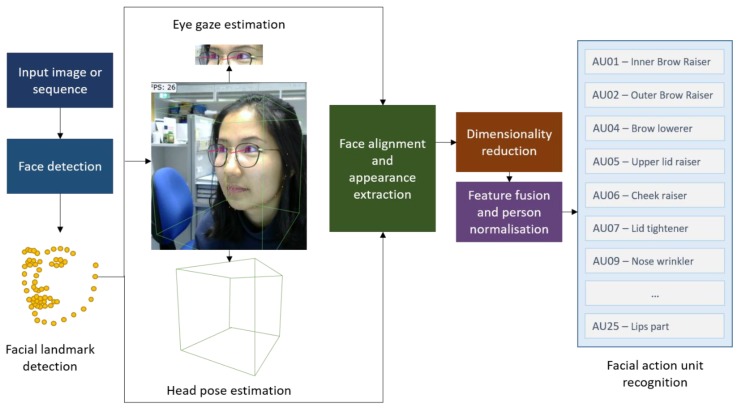
OpenFace architecture based on [120].

The primary step of face expressions recognition is face detection by detecting eyes, mouth and nose as reference points [116]. Action Units (AU) are commonly used to classify the changes in facial features [117]. AU-based recognition is a group of basic face muscles actions, with each action represented by a number. For instance, AU01 is inner brows raise and AU07 is lower eyelids raise [118]. The neural network is used for detection, tracking and further analys of emotional or cognitive states [119]. One frequently used open source software is *OpenFace* which supports various advanced functionalities such as real-time analysis and does not require calibration [120]. Figure 14 presents the facial behaviour analysis architecture of OpenFace.

For face detection and facial landmark detection, Conditional Local Neural Fields (CLNF) are used in this software. CLNF exploit advanced patch experts that capture the variations of local appearance. This model works well on webcam and allows real-time processing. Gaze estimation is additional feature that makes OpenFace different from other software [120]. The results from OpenFace are AUs which needs a further analysis for emotional or cognitive states. Different combinations of AUs are associated to specific emotional states. Basic emotions comprise of happiness, anger, sadness, fear, surprise, neutral and disgust. In addition, compound emotions include a combination of two basic emotions [121].

## 4. Machine Learning in Estimation Modules

The uses of each sensor and the performance characterisation were described in previous sections. This section discusses the techniques to make use of the collected data from the sensors to estimate cognitive states, as in the estimation module in CHMI^2^ framework. Particular importance lies in the fusion of the data from multiple sensors because, as discussed in Section 1, the various neurophysiological variables are indicative of different cognitive states and are characterised by different uncertainties and characteristic times. The fusion of data from multiple neurophysiological sensors to estimate cognitive states can follow three fundamental approaches: (A) independently estimating cognitive states from each sensor then fusing these estimates; (B) cognitive state estimation based on a fused pool of extracted features from each sensors, and (C) using data from one or more sensors to extract more/different information from another sensor and/or for sanity checks. Approach (A) supports the use of simpler legacy statistical methods and data analytics techniques for the data fusion, but is less robust as individual observed extracted features can be caused by multiple combinations of cognitive states. Approach (B) is more reliable than (A) as the simultaneous observation of different features mitigates the ambiguity in cognitive state estimation, however it requires more complex data fusion techniques to account for a high number of frequently nonlinear relationships. Approach C is potentially the most reliable and robust, however requires a much deeper understanding of neurophysiological processes, partly not yet achieved at this stage. A well-designed multi-sensor system yields a minimal uncertainty in the cognitive state estimation, hence supports a more reliable and robust inference of cognitive states to drive system reconfiguration. The CHMI^2^ estimation module emulates the mathematical correlations between the sensed neurophysiological variables and the cognitive state variables that are passed to the adaptation layer. However, in addition to the limited consent in the literature on the nature of the mathematical correlation between neurophysiological measurements and cognitive states, differences in individual characteristics can be very significant in terms of neurophysiological response and maximum endurable workload and fatigue levels. These important factors prompted researchers in this domain to explore suitable classification techniques from statistics or computer science (machine learning), which can support both the determination of the overall correlation and also their fine-tuning to the particular conditions of the individual. The most commonly adopted methods to estimate human cognitive states from psycho-physiological data include: artificial neural networks [64,67], fuzzy systems [122,123], discriminant analysis [71,124], Bayesian models [53,125,126], SVM [127,128], and committee machines [82,129].

Artificial neural networks attempt to emulate the workings of the human neurons, each acting as a node performing simple functions, but which can be combined in a very large number of neurons. The connections between nodes are governed by weights, which are to be tuned during a preliminary training phase, allowing the machine to ‘learn’. Fuzzy logics attempt to mimic the human brain in software, employing logical reasoning to make inferences from observed states based on how close to the expectation is a recorded value. Expert knowledge is stored in an “if-then” rules database which maps a fuzzy set of input data to a fuzzy set of output data. The linguistic structure of the rule base offers a primitive explanation of the system’s reasoning from both the researcher and end-user perspectives; however, the usability and significance of this explanation is quickly lost when increasing the number of inputs and outputs and the complexity of the fuzzy membership functions. Neural-Fuzzy Systems (NFS) [130,131], are conceived to combine the advantages of both artificial neural networks and fuzzy inference systems. NFS are an effective method of determining the unknown correlations in presence of high measurement uncertainties and show much better repeatability and technological maturity compared to other techniques.

### 4.1. Neuro-Fuzzy Inference Concept

**Figure 15:**
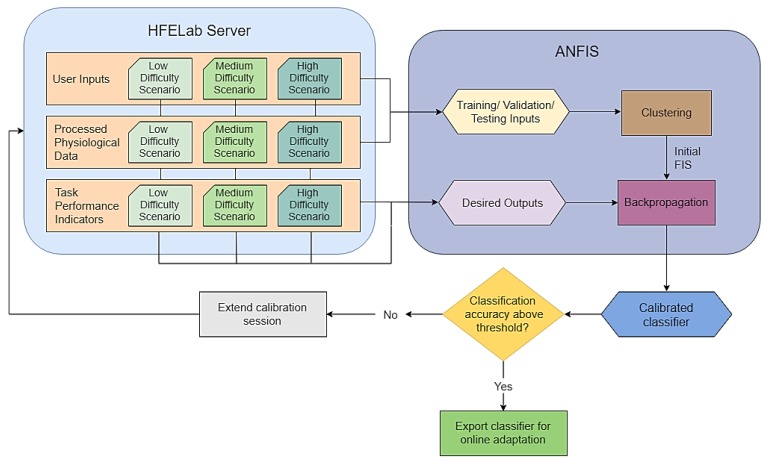
Offline calibration of CHMI^2^ inference system.

The estimation module of the CHMI^2^ infers the cognitive states (e.g., workload, fatigue, attention, etc.) based on a combination of neurophysiological, environmental and task-specific input data streams following approach (B) described in the previous paragraph. A neuro-fuzzy implementation allows these input-output relationships to be described through fuzzy IF-ELSE rules, which provides greater diagnosticity and transparency than other machine learning methods. Fuzzy systems provides some flexibility in adapting the system parameters to individual users so that the correlations exploited by the CHMI^2^ are unique to different individuals and their daily neurophysiological/mental state. Fuzzy logic provides a simple structure to the classifier and support a greater degree of result interpretability when compared to other machine learning approaches such as deep learning. The fuzzy logic is encoded in a simple neural network, allowing the fuzzy system to be fine-tuned using offline or online learning. The offline calibration of CHMI^2^ inference system is presented in Figure 15.

Fuzzy logic captures well some aspects of the ambiguity and subjectiveness of human thinking, and is used to model specific aspects of uncertainty. Unlike probability, which expresses the likelihood of an event’s occurrence, fuzzy logic expresses the degree of truth of that event occurring. As an example, the MWL of an operator can be expressed in the following manner:

- MWL has a probability of 0.15 to be high and 0.85 to be medium.
- MWL is high to a degree of 0.15 and medium to a degree of 0.85.

The categories of high, medium and low can be expressed by fuzzy sets. The degree to which an observed event belongs to each category is described by the membership value of each fuzzy set.

### 4.2. Fuzzy Sets

**Figure 16:**
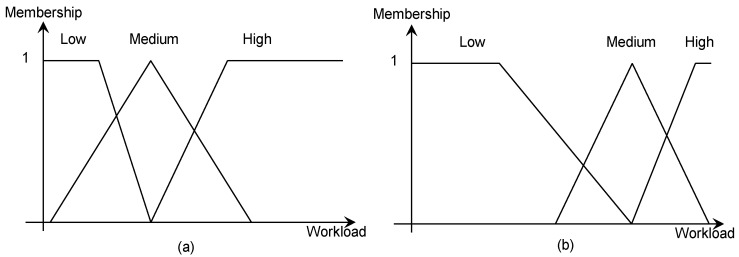
Fuzzy sets associated to different workload tolerance of individuals. Compared to (**a**), (**b**) shows an individual with a higher tolerance for high workload conditions.

In the context of the CHMI^2^ research, fuzzy sets are used to describe specific characteristics of the human user, such as their neurophysiological or cognitive features. Fuzzy rules are then used to describe the relationship between the user’s neurophysiological and cognitive states, such as in the case of HR, BLR, Dwell Time (DT), MFA in:

R1: IF [(HR is Low) AND (BLR is High) AND (DT is High)] THEN [(MFA is High)]
R2: IF [(HR is High) AND (BLR is Low) AND (DT is High)] THEN [(MWL is High)]

and so on. The parameters of the fuzzy sets (such as a set’s centres and spreads) are assumed to differ across individuals. For example, a novice operator might have a workload tolerance as characterised by the fuzzy set shown in Figure 16a while an experienced operator might have a workload tolerance as characterised by the fuzzy set shown in Figure 16b.

A suitable fuzzy inference system for CHMI^2^ then needs to specify (1) the number for fuzzy sets used to represent each input feature, (2) the type and parameters of the membership function used to describe each fuzzy set, (3) the rules which characterise the relationship between the input features and output cognitive states, as well as (4) the inference method employed. Figure 16 illustrates typical Trapezoidal membership functions—“fuzziness” derives from the overlaps in the membership functions.

### 4.3. Neuro-Fuzzy Networks

Neural fuzzy systems allow the structure of fuzzy inference systems to be expressed as a neural network. Neural-fuzzy systems networks afford a high degree of flexibility in optimising the parameters of the fuzzy inference system, given suitable training data. The architecture of a basic neural-fuzzy network typically contains the following layers:

- Input layer: each node passes the input values to the next layer.
- Antecedent layer: each node fuzzifies the inputs using a membership function. The node output is the fuzzy set membership for a given input parameter.
- Rule layer: each node combines the fuzzified inputs using a fuzzy AND operator. The node output is the rule firing strength. For example, *K* Sugeno-type rules, where the rules can be formulated as [9]:
  Rule *k*: If *x*_1_ is *A*_1*n*_ and *x*_2_ is *A*_2*n*_ and … and *x_i_* is *A_in_* then *f_j_* = *p_k_*_0_*+ p_k_*_1*×*1_ + *p_k_*_2_*x*_2_ + … + *p_ki_x_i_*
  where *A_in_* is the *nth* membership function of input *x_i_*, *f_j_* is the output node function associated with output *j* and *p_ki_* denotes the coefficients of this node function for rule *k* and input *i*.
- Consequent layer: each node combines the fired rules using a fuzzy OR operator. The node output is the membership value of the output parameter.
- Output layer: each node acts as a defuzzifier for the consequent nodes to obtain a crisp output.

### 4.4. Membership Functions

**Figure 17:**
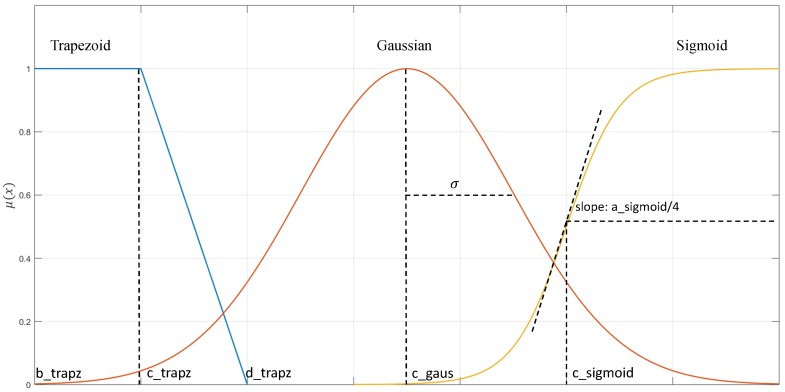
Membership function: Trapezoid, Gaussian and Sigmoid.

**Figure 18:**
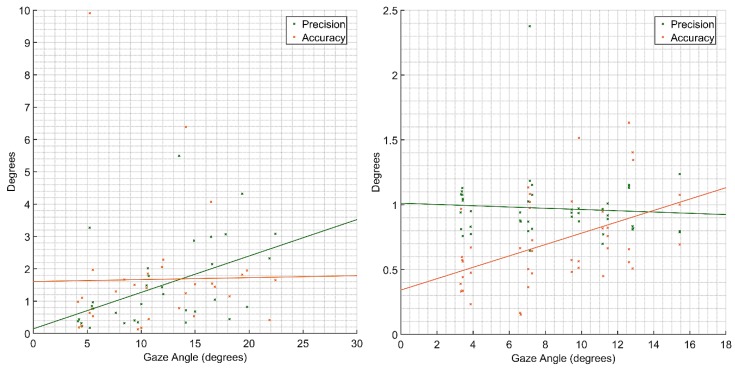
Precision and accuracy of gaze angle. **Left**: a wearable eye tracker. **Right**: a remote eye tracker.

As conceptually depicted in Figure 17, the fuzzy set is characterised by its membership functions. These can assume various forms, which yield different advantages and disadvantages. The most common types include Trapezoidal, Gaussian, and Sigmoidal functions, which are described below. Additionally, Figure 18 only shows an example of some different membership function types but in the real system, these three different types cannot be used together.

(1). Trapezoidal membership function

The trapezoidal membership is defined by four different parameters (a, b, c, d), with *a* < *b* < *c* < *d*, as:

(32) $$\mu \left(x\right)=\{\begin{array}{cc} 0, & x\leq a\\ \frac{x-a}{b-a}, & a\leq x\leq b\\ 1, & b\leq x\leq c\\ \frac{d-x}{d-c}, & c\leq x\leq d\\ 0, & x\geq d \end{array}$$

(2). Gaussian membership function

The Gaussian membership function is defined by parameters (c, σ) as:

(33) $$\mu \left(x\right)=\exp\left[-\frac{{\left(x-c\right)}^{2}}{2\sigma^{2}}\right]$$

(3). Sigmoidal membership function

The sigmoidal membership function is defined by parameters (a, c) as:

(34) $$\mu \left(x\right)=\frac{1}{1+\exp\left(-a\left(x-c\right)\right)}$$

## 5. Sensor Performance Characterisation

Sufficient accuracy and reliability of neurophysiological measurements is essential to successfully realise the human-machine system concepts described in Section 1. The uncertainties from sensors are discussed in this section. Moreover, this section also presents the performance characterisation methodologies for two neurophysiological sensors that are being used to support the development of the CHMI^2^ concept: eye-tracking sensors and wearable cardiorespiratory sensors. Lastly, the propagation of uncertainty from eye activity and cardiorespiratory from inference system are discussed.

### 5.1. Eye-Tracking Sensors

The eye tracking performance is commonly characterised by three parameters: sampling frequency, accuracy and precision. Sampling frequency is number of measurements per second (Hz). Accuracy is the difference between true eye position and measured position (in°). Precision is the measured gaze consistency (in°). The precision of each cluster is calculated based on Equation (5) and the mean accuracy computed from Equation (6):

(35) $$\theta_{RMS}=\sqrt{\frac{\theta_{1}^{2}+\theta_{2}^{2}+\ldots +\theta_{n}^{2}}{n}}$$

where $\theta_{i}$ denotes the angular distance of the *i*-th sample:

(36) $$\theta_{acc}={\bar{\theta }}_{i}-\theta_{i}^{*}$$

where ${\bar{\theta }}_{i}$ is the mean location of all the points in cluster *i*, while $\theta_{i}^{*}$ is the cluster’s actual location.

Additionally, the uncertainty analysis can be studied from the measured scene camera’s Field of View (FOV). FOV is geometric distance from the object to the camera. The propagation of uncertainty given by:

(37) $$\sigma_{FOV}=\;\frac{\sqrt{\sigma_{l}^{2}+\;{\left(\frac{l}{d}\right)}^{2}\sigma_{d}^{2}-\frac{2l}{d}\sigma_{ld}}}{d\left[1+{\left(\frac{l}{2d}\right)}^{2}\right]}$$

where $\sigma_{l}$ is uncertainty from the object measurement, $\sigma_{d}$ is uncertainty from distance measurement, *l* is the object known dimensions and *d* is the distance from the object to the camera. $\sigma_{ld}$ is the covariance of measured distance. In order to get a conservative $\sigma_{FOV}$, $\sigma_{ld}$ is assumed to be zero.

In a recent study, we investigated the experimental characterisation of remote and wearable eye-tracking sensors in detail [9]. The study covered the mentioned three parameters and the uncertainty of FOV. The methodology for static performance is that subjects fixate on 16 static different points spacing around with larger gaze angles. The precision and accuracy of gaze angles for both type of sensors are presented in Figure 18. The accuracy of wearable sensor is consistent with a value of 1.7° across the gaze angle ranges while precision of the remote sensor is consistent with a value of 1° across the gaze angle ranges.

**Figure 19:**
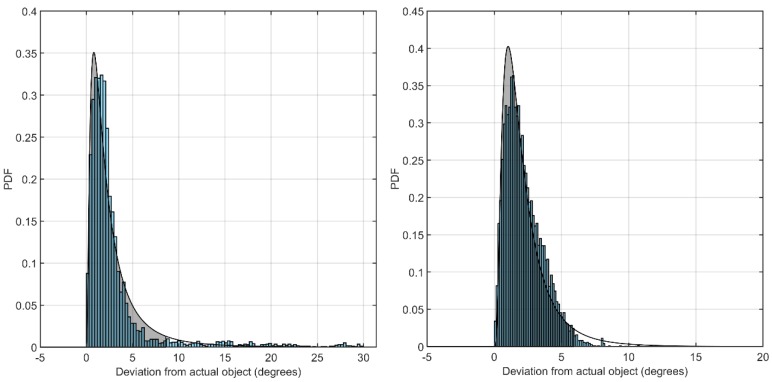
Fitting curve of lognormal distribution with 95% of all gaze points including in shaded area. **Left**: wearable eye tracker. **Right**: remote eye tracker.

The dynamic performance was studied by having participants track a moving object along a given trajectory. Human error had a significant influence on the tracking performance since the results revealed gaze trails that were leading or lagging the moving object, leading to poorer performance compared to the static case. The 2-sigma accuracy with approximately 95% of all gaze points (Figure 19) was calculated to 8.6° and 5.9° for the wearable and remote eye tracker, respectively.

Although not addressed by this study, blink rate performance is also an important aspect for human-machine systems. The true blink rate can be quantified by manual counting from the recorded video of the sensors. Using this, the blink rate error can then be calculated by:

(38) $$BLR_{error}=\;\frac{false\;positive+false\;negative}{total\;no.\;of\;blink\;}$$

### 5.2. Cardiac Sensors

The performance criteria for cardiorespiratory sensors are validity, reliability and sampling frequency. Validity is the difference between the baseline and measured data. Reliability is the consistency of the results within the variable. Sampling frequency is the same as described in Section 4.1. A common methodology of characterising the performance of such sensors is to compare the measurements of clinically validated sensors with the sensor of interest [29,30]. The validity can be calculated given by Equations (9) and (10):

(39) $$RMS_{error}=\sqrt{\frac{1}{n}\sum_{i=1}^{n}{\left(x_{i}^{ref}-x_{i}^{measured}\right)}^{2}}$$

where *n* is the number of data and $x_{i}^{ref}$ and $x_{i}^{measured}$ are the reference and the measured values respectively. The Correlation Coefficient (CC) is given by:

(40) $$CC=\;\frac{n\left(\sum^{\;}xy\right)-\left(\sum^{\;}x\right)\left(\sum^{\;}y\right)}{\sqrt{\left[n\sum^{\;}x^{2}-{\left(\sum^{\;}x\right)}^{2}\right]}\left[n\sum^{\;}y-{\left(\sum^{\;}y\right)}^{2}\right]}$$

where *n* is the number of data and *x* and *y* are the reference datum and the measured values respectively.

A comparative evaluation of the wearable sensor data against a clinically validated device is pursued in the characterisation experiment. For heart activity, a clinically validated ECG sensor is used as baseline while a spirometer is used for respiratory monitoring. Two types of exercises are conducted, involving high physical and mental workload respectively. The physical exercise can be subdivided into three parts: pre-exercises, exercise and post-exercise, lasting one minute, three minutes and one minute, respectively. In the mental exercise, three sub-sections are carried out, including *Mental Rotation*, *Hampshire Tree Task* and *N-back Task*. Consistently with the raw signal treatment used in a majority of cardiac monitoring devices, Butterworth low-pass filtering is applied to the collected data, so that signals higher than a selected cut-off frequency are lessened. This type of low-pass filter is the most consistent noise removal process for most neurophysiological measurements, as it removes any high-frequency content, which cannot be physically generated by biological processes. Such process increases the accuracy of the neurophysiological measurement and is included in the experimental characterisation as there would be little interest in characterising the raw data.

Table 9 presents the comparison of HRV measurements between a commercial wearable device and a clinical-validated one. Overall, the RMS error is lower than 0.1 which means that there are minimal errors between the two devices. Concerning the *CC*, the result shows good correlation (i.e., *CC* ≥ 0.75).

### 5.3. EEG Sensors

**Figure 20:**
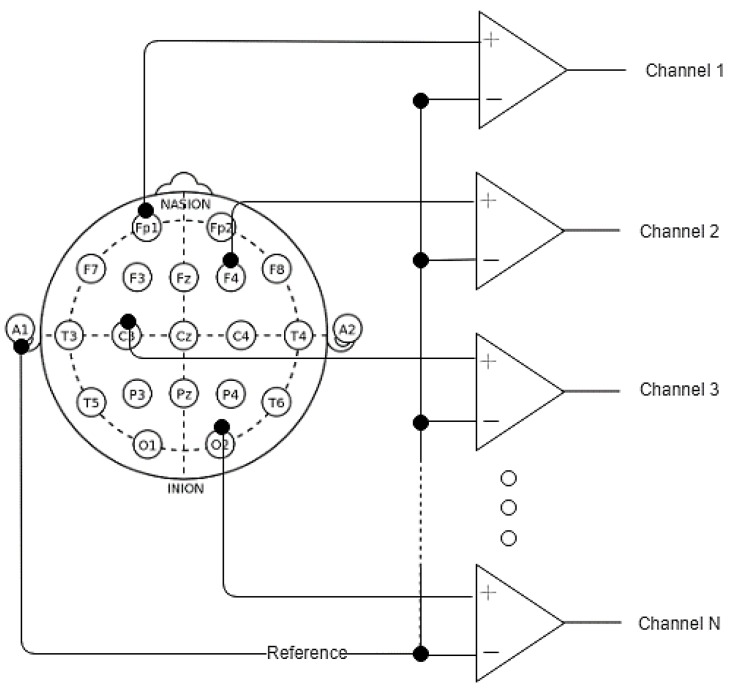
Referential montage.

The EEG performs measurements by using differential amplifiers, as schematically illustrated in Figure 20. The circuit functions by comparing two input voltages from two different electrodes and giving an output voltage that amplifies the difference between the two voltages and cancels out common voltages. This is described by the equation below. The input signals can be compared in various arrangements referred to as montages. A commonly adopted layout is the referential montage, where all channels are compared with a common reference:

(41) $$V_{out}=\;A\left(V_{in}^{+}-V_{in}^{-}\right)$$

**Figure 21:**
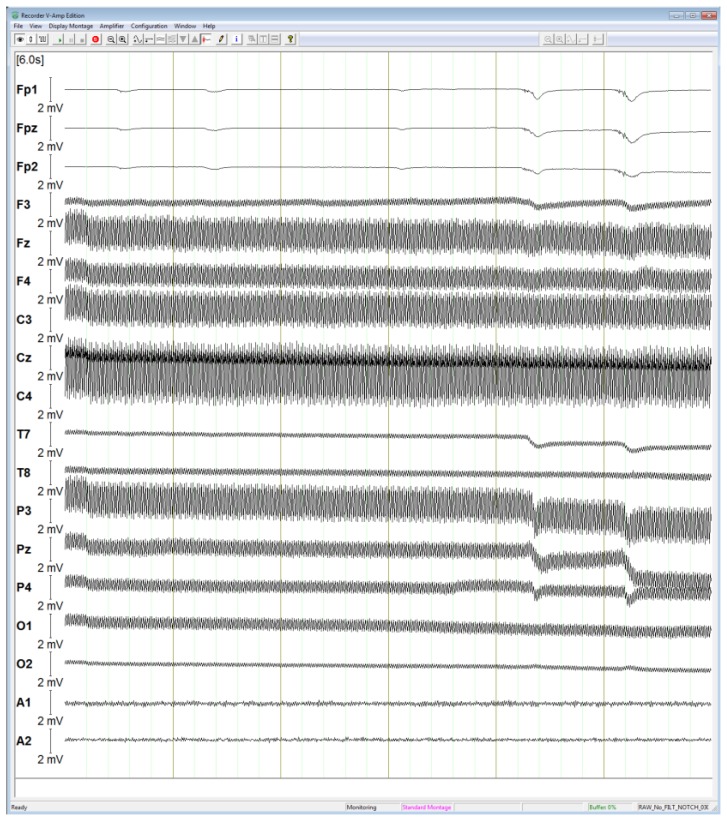
Raw EEG signal with excessive noise.

The raw EEG signal measured is displayed in Figure 21. The shown measurement is performed using 16 data electrodes, one reference electrode and one ground electrode. The placement of electrodes is described following the 10–20 international system. Generally, the amplitude of the EEG signals is up to 100 µV [101], however the amplitude of raw signals in the figure is much larger as they are affected by a large interference from the mains power (240 V/50 Hz), which is discussed in Section 3.3. Some blink artifacts are observed on several channels and particularly Fp1, Fpz and Fp2 but since these raw measurements underwent no filtering, they are contaminated with a high interference from the mains power.

**Figure 22:**
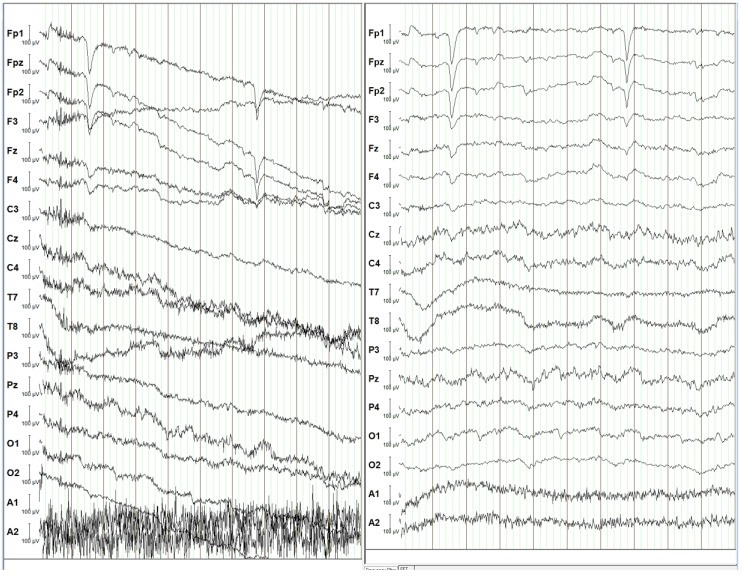
**Left**: EEG signal with notch filter only. **Right**: EEG signal with notch, low pass and high pass filters.

By applying suitable filters to the raw data, it is possible to extract the desired EEG signals, as shown in Figure 22. Both windows in the figure display the EEG signals that are passed through a 50 Hz notch filter. However, in addition to the notch filter, the signals in the right window are also processed through a 0.1 Hz high pass filter and a 30 Hz low pass filter. The raw data gathered from the EEG electrodes are processed with software filters for the high pass, low pass and notch filters, where the digitized signals amplitude of the corresponding frequencies are reduced. The software uses a Butterworth filter, with a slope of 12 dB/octave for the high and low pass filters. While the amplitude of signals in both windows remains mostly within 100 µV, the signal in the left one is noisier compared to the right window. Most importantly, the lack of the low-frequency high pass filter causes signals in the left window do drift from the initial value. Henceforth, the filtered measurements in the right window are the closest estimation of the electrical activity originated in the subject’s brain. Applying the low pass and high pass filters eliminates the undesired components, as the signals of interest mainly lie within 0.1 Hz to 30 Hz [132]. After frequency filtering, blink and other movement-related artifacts are however still imbedded in the signal. These can most clearly be seen in the first three channels as dips in the signal. Such neurophysiological artifacts are undesirable as these electrical signals do not originate from within the brain [101,103]. Subsequent signal processing focusses on the frequency domain as different cognitive states can be determined from the subject by using a power spectrum analysis [132].

### 5.4. Propagation of Uncertainty

This section describes the analysis of uncertainty propagated through the estimation module of CHMI^2^. The methodology of this analysis was introduced in [9]. Adaptive Neuro-Fuzzy Inference Systems (ANFIS) [133] are considered in this analysis. The propagation of uncertainty is calculated through five layers. The final uncertainty from the output layer is given by:

(42) $$\sigma_{y_{j}}=\sqrt{\sum_{k^{*}}{\left(f_{i}\cdot \sigma_{{\bar{w}}_{k}}\right)}^{2}+{\left(\frac{\prod_{i^{*}}\mu_{A_{\hat{i}}}\left(x_{\hat{i}}\right)}{\sum_{k^{*}}\prod_{i^{*}}\mu_{A_{\hat{i}}}\left(x_{\hat{i}}\right)}\right)}^{2}\cdot \sum_{\hat{i}\neq 0}{\left(p_{k\hat{i}}\cdot \sigma_{x_{\hat{i}}}\right)}^{2}}$$

where, *i* represents input, *j* represents output, $f_{i}$ and $p_{k\hat{i}}$ are known from *K* Sugeno-type rules describes in Section 4.3, *k* is number of rules, $\mu_{A_{\hat{i}}}$ is membership function from input layer which Gaussian membership function (Equation (33)) is used, $\hat{i}$ denotes an iterator, $\sigma_{x_{\hat{i}}}$ is known uncertainty input and $\sigma_{{\bar{w}}_{k}}$ is the uncertainty associated with normalisation layer, which was discussed in detail in [9].

In MWL case study, the participant has to accept new arrival or departure traffic from upstream ATM sectors by himself in this event. The ANFIS-based system was prompted to identify the correlation between the HR and BR in mental workload condition:

1. If HR is high and BR is low, then MWL = 1
2. If HR is mid and BR is mid then MWL = 0.5
3. If HR is low and BR is high, then MWL = 0.1

MWL is quantified by the number of aircraft in the scenario. Table 10 details the cluster centres for the participant.

**Figure 23:**
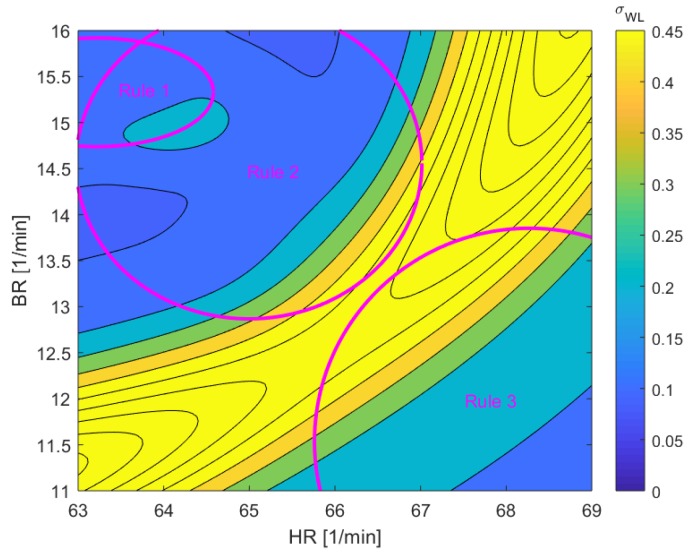
ANFIS inference uncertainty from breathing rate and heart rate for mental workload.

The uncertainty input of HR σ_HR_ = 5.5 min^−1^ and BR σ_BR_ = 1.6 min^−1^ are applied to define the output uncertainty of the fuzzy system which is illustrated on Figure 23. The high uncertainty region occurs mostly where the rules get conflicted.

## 6. Aerospace Applications

In recent years, the RMIT Cyber-Physical Systems (CPS) Group has conducted several Research and Development (R&D) projects supported by the Australian Government and high-calibre industry partners in the area of Cognitive Human-Machine Systems (CHMS) and Neuroergonomics for avionics, Air Traffic Management (ATM), and defence/space systems. Numerous lessons were learned from contemporary human factors/ergonomics and medical studies demonstrating that human performance in complex and demanding tasks is affected by a variety of neurophysiological and environmental factors, which can be readily measured and analysed using advanced cyber-physical system architectures, including sensor networks and Artificial Intelligene (AI)/ML techniques.

Neurotechnology is a promising field of research attracting increased attention and resources. Australia is an emerging player in this field, with several ongoing aerospace/defence and transportation R&D initiatives and with various new-entrant enterprises, which have been founded to develop neurotechnologies predominantly for precision/preventive medicine and advanced therapeutic applications. In parallel with evolutions driven by a deeper understanding of the human brain and its functions, intelligent automation and trusted autonomy are being introduced in present day cyber-physical systems to support diverse tasks including data processing, decision-making, information sharing and mission execution.

Due to the increasing level of integration/collaboration between humans and automation in these tasks, the operational performance of closed-loop human-machine systems can be enhanced when the machine monitors human stressors and cognitive states and adapts to them in order to maximise the effectiveness of the Human-Machine Teaming (HMT). Recent technological developments have led to neurophysiological observations becoming an increasingly reliable methodology to evaluate human cognitive states (e.g., workload, fatigue and situational awareness) using a variety of wearable and remote sensors. The adoption of ad-hoc sensor networks can be seen as an evolution of this approach, as there are notable advantages if these sensors collect and exchange data in real-time, while their operation is controlled remotely and synchronised.

### 6.1. Single Pilot Operation and Unmanned Aircraft Systems

Single Pilot Operations SPO are currently possible only in the military, general aviation and business jet domains, whereas a crew of at least two pilots is currently mandated for airline transport aircraft, i.e., the ones certified under the so-called *Part 25* of the various national airworthiness policies. Due to the substantial growth in commercial air travel demand and an aggravating global shortage of qualified airline pilots [134], SPO is becoming an attractive option for airline transport aircraft within the next two decades [135]. However, single pilot operated transport aircraft are faced with great challenges, as the pilot on board may become incapacitated, thus resulting in potentially fatal accidents. Moreover, SPO may in certain conditions pose an excessive cognitive demand on the single on-board pilot, as the capacity for cognitive work is limited in humans. To address these challenges, SPO concepts include novel avionics systems such as a Virtual Pilot Assistant (VPA) and a Ground Pilot (GP) [136]. The combination of a VPA and GP provides a promising solution to perform the functions normally accomplished by the Pilot Not Flying (PNF) in airline transport aircraft. The VPA shall support advanced and highly automated surveillance, communication and flight management capabilities, including adaptive task allocation through CHMI^2^. For instance, the VPA interfaces with the Separation Assurance and Conflict Avoidance (SA&CA) function of the Next Generation Flight Management System (NG-FMS), which supports autonomous flight planning, deconfliction and real-time re-optimisation capabilities. Additionally, the VPA system shall promptly detect an incapacitation event, thus triggering a reallocation of all tasks and giving the VPA autonomous control while at the same instant alerting and transferring the human control authority to the GP. The VPA shall therefore monitor the on-board pilot using non-intrusive sensors. Early VPA research and experimentation will include a variety of monitoring devices for measuring central nervous parameters, eye movements, cardiorespiratory parameters, facial expression and voice patterns.

**Figure 24:**
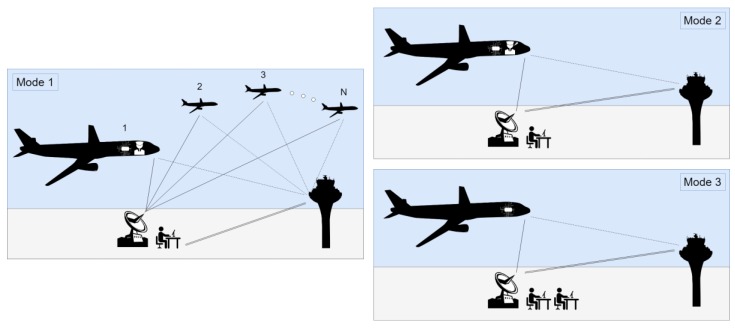
Integrated Air-Ground Concepts of Operation for SPO and UAS remote control.

SPO involves various modes of operation, as discussed in [137] (see Figure 24). The first and nominal mode includes the single on-board pilot and the VPA cooperating regarding the decision making and flying tasks, while the GP provides dispatch information and communication with Air Traffic Control (ATC).

In nominal operation the GP will act in this role for a dynamically varying number of SPO aircraft determined by the adaptive CHMI^2^ framework. Hence, if a GP is under high or low cognitive workload the number of aircraft assigned will be adjusted. How the GP will maintain ongoing situational awareness of, and switch context amongst, the assigned aircraft are important issues for the CHMI^2^ to address. Under a circumstance where the on-board pilot is under high workload, such as take-off, landing and unforeseen events, a GP will be specifically allocated to the aircraft, so that the GP then provides dedicated assistance. Hence the GP will act as a ground located PNF, where both human operators would continuously monitor the instruments, radio communication and perform crosschecks when notified about changes by the VPA. In the third mode the on-board pilot has become partially or fully incapacitated. Here the VPA has full autonomy of the aircraft until a team of two GP take over control authority and supervise the aircraft to a safe landing at the nearest airport available.

**Figure 25:**
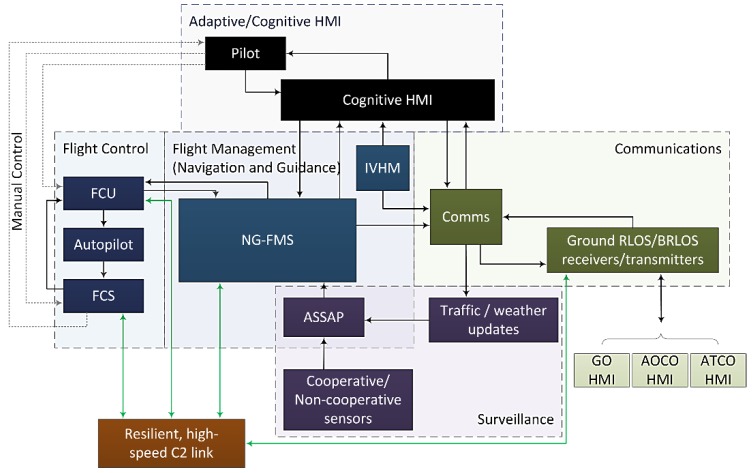
VPA system architecture from [136].

The main components of the VPA include the flight management, communication, surveillance and CHMI^2^ modules, the corresponding system architecture can be seen in Figure 25 [136]. The CHMI^2^ is a crucial component of the VPA system, providing the necessary reductions in workload as well as incapacitation-detecting capabilities that will support the case for SPO certification. The CHMI^2^ assists the pilot with several intelligent functions such as information management, adaptive alerting, situation assessment as well as dynamic task allocation. The combination and the interactions between these modules to support the on-board pilot and the GP is the core VPA. The system has some important capabilities that includes a reliable, secure and high-speed Command and Control (C2) link, where the GP can take direct control of the aircraft from the Ground Control Station (GCS) similarly to a medium-large UAS, and an Airborne Surveillance and Separation Assurance Processing (ASSAP), which provides autonomous SA&CA.

### 6.2. One-to-Many and Air Traffic Management

The One-to-Many (OTM) concept refers to a situation in which multiple UAV are controlled and commanded by a single operator. As of today, OTM operations are still challenging for the human operator since they can induce an excessive mental workload due to cognitively demanding and time-critical tasks [138]. The design of HMI^2^ for the supervisory control of multiple UAV is therefore a main area of research. The HMI^2^ for supervisory control shall provide suitable information and level of automation to maintain the cognitive states of the human operator within a desirable range [139]. Hence, the application of the CHMI^2^ concept is particularly promising for OTM ground control stations. Applying the CHMI^2^ concept, the real-time adaptation in HMI^2^ ground control station is driven by the human operator’s cognitive states: Mental Fatigue (MFA), Mental Workload (MWL) and Situation Awareness (SA) to enhance decision making and mission performance [140,141].

The concept of a single human operator coordinating multiple assets is key to ATM, which however deals only with a limited subset of functions: deconfliction, advisory/information/alert services and traffic flow optimisation. Air Traffic Control (ATC), a major component of ATM is deemed one of the most demanding cognitive tasks for human beings as it involves complex and time-critical situation assessment and decision-making related to multiple aircraft. The MWL of Air Traffic Controllers (ATCo) has been the main focus of several studies to improve the safety and efficiency of the ATM system [61,142]. In order to quantify the MWL of ATCos, early studies used Electrocardiograph (ECG) devices to monitor sinus arrhythmia as a measure [143]. One main limitation of this approach is that the ATC task requires verbal communication, which affects the sinus arrhythmia measurements. Thus, various other sensors were used to investigate MWL of ATCos. The neurophysiological sensors include ECG, eye tracking and Electroencephalography (EEG) devices [57]. Most of the neurophysiological parameters changed as expected during MWL increasing: Heart Rate (HR), Breathing Rate (BR), Heart Rate Variability (HRV), and blood pressure. Likewise, a suitable set of neurophysiological sensors are continuously studied with respect to MWL of ATCos feasibility and sensitivity.

The complexity of the ATC task correlation with spectral power was studied by EEG [72]. Additionally, fNIRS, one of neuroimaging sensors, also uses to study MWL in ATC mission; certified professional controllers under realistic scenarios with emergent and typical condition were monitored. The results show the relation of fNIRS and MWL in real scenarios [92]. Apart from MWL, Situation Awareness is one of the key important cognitive states for ATCos. The eye tracking sensor was started to monitor operators’ eye activities since visual workload is the main causes of MWL for ATCos [144]. However, the use of a single sensor is not optimal. Hence, the deployment of neurophysiological sensors to operational settings could help evaluate the cognitive state of staff assigned to perform critical tasks and contribute to improving the safety and efficiency of human-machine systems [145]. The CHMI^2^ concept was applied successfully to the ATM domain, which has the important advantage of involving reasonably standardised scanning patterns and phraseology, benefiting eye-tracking and voice pattern analysis techniques respectively.

### 6.3. Space Applications

The CHMI^2^ concept and the associated neurophysiological sensor network implementations have a clear potential in the space application domain. Since the NASA Mercury, Gemini, and Apollo programs, sensor systems have been used to collect astronaut neurophysiological data to identify and plan for support activities that counteract the effects of degraded performance on mission safety [146]. Likewise, during the shuttle era, an ECG system known as the Operational Bioinstrumentation System (OBS) was used to monitor astronaut neurophysiological health during launch and re-entry phases [147]. Today, the International Space Station (ISS) contains the most comprehensive neurophysiological sensor system known as the Crew Healthcare Systems (CHeCs) and is the primary means of astronaut neurophysiological monitoring [148]. The CHeCs is comprised of a suite of neurophysiological sensors including blood pressure (BP), electrocardiograph (ECG), and heart rate monitoring (HRM) systems. The measurements from the CHeCs system are solely used to infer the real-time physical health state of astronauts during periodic fitness and health evaluations, as well as to support scientific experiments on cardiovascular physiology. Similarly, neurophysiological sensors are incorporated into the spacesuits used during Extra-Vehicular-Activities (EVA) as well as other forms of advanced life support. As of today, neurophysiological observations from the CHeCS and EVA spacesuit systems are not used to infer astronaut cognitive state, but rather via a self-administered neurocognitive assessment [149]. The Spaceflight Cognitive Assessment Tool for Windows (WinSCAT) is a time constrained cognitive battery comprised of well understood neurophysiological tests including verbal and visual memory, mental arithmetic, sustained attention and spatial imagery [150]. The test is performed approximately on a monthly basis and is assessed against the baseline performance of the individual determined in pre-flight conditions to provide a “fitness-for-duty” assessment as opposed to real time monitoring.

In combination with state-of-the-art research into the development of comprehensive, wearable and non-invasive neurophysiological sensors such as NASA Lifeguard system [151], existing space-based neurophysiological sensor networks and cognitive assessment tools form in part the underlying sensor framework to address the evolution towards human-machine systems based on real time cognitive assessment for safety and mission critical systems. Additionally, the success (although limited) of previous research [149] into inferring cosmonaut and astronaut cognitive state through voice pattern analysis should be capitalized on, as current state-of-the-art neurophysiological sensor networks show promise in removing previously encountered limitations in the system’s ability to deterministically distinguish between stress or emotional arousal in recorded voice. Most importantly, and in direct alignment to the requirements set in NASA’s Bioastronautics roadmap [152], the employment of closed loop human machine systems and associated cyber-physical sensor networks will play a key part in meeting the current challenges to mitigate human factors risks with low earth orbit (LEO) space flight along with new and exciting challenges associated with lunar and long-term planetary missions.

## 7. Conclusions

This article addressed the increasingly important role of sensor networks in aerospace cyber-physical system applications, focusing on the sensors used to enhance human-machine teaming, such as those enabling the implementation ofCognitive Human-Machine Interfaces and Interactions (CHMI^2^) system concepts. Many safety-critical tasks are inherent in aerospace applications such as Air Traffic Management (ATM) and a reliable monitoring of the human operator will be highly instrumental in the future due to the severe consequences of reduced performance. On the other hand, space applications currently mainly use sensor networks for medical monitoring purposes. However, human-machine interactions in space are expected to evolve considerably in the future. The main aspects associated with neurophysiological sensors were described: the state of art, neurophysiological parameters and their relationship to human cognitive states. Depending on the adopted neurophysiological measures, the minimum performance requirements are different. Moreover, some of the measures can be affected by the operator’s level of training and experience such as in the case of Heart Rate (HR) and Heart Rate Variability (HRV). The summary of cognitive states shows that the combined use of diverse sensors in a network can improve the reliability and accuracy of cognitive states estimation with respect to using only single measures, since the change in one measure is typically correlated to several cognitive states. It is essential that the suite of sensors records neurophysiological data reliably and accurately. This paper also briefly discusses the characterisation of an eye tracking and cardiorespiratory sensor being used in the CHMI^2^ framework. The results show that the sensors have an adequate performance for use in the framework.

## Figures and Tables

**Table 1:** Eye activity metrics adapted from [3] includes Equations (1)–(13).

Parameter	Description	Derived Metrics	Equation	Equation Number
Fixation	The state of a gaze that is focused (fixated) on an object.	Fixation (duration, frequency, count)	$f_{n}:\sqrt{{\left(x_{max}-x_{min}\right)}^{2}+{\left(y_{max}-y_{min}\right)}^{2}}\;<\;D_{max}$ $\forall \;t\in \left[t_{0},t_{fn}\right]$	(1)
		Time to first fixation	$\;T_{FirstFixation}=t\left(f_{1}\right)-t_{0}$	(2)
Saccade	Small, rapid, involuntary eye movements between fixations, usually lasting 30 to 80 ms.	Saccadic length/amplitude, frequency	$s_{n}:\;\;\;v\left(t_{s_{n}}\in \left[t_{i},t_{j}\right]\right)\;\geq 30\;{}^{\circ}/s$ with $t_{i}+30\;ms\;\leq t_{j}\leq t_{i}+80\;ms$ and $v=\;\sqrt{{\left(\frac{dx}{dt}\right)}^{2}+{\left(\frac{dy}{dt}\right)}^{2}}$	(3)
		Saccade velocity (mean/peak)	${\bar{v}}_{s_{n}}=\;v\left(t_{s_{n}}\right)$ $v_{max,\;s_{n}}=\max\left(\;v\left(t_{s_{n}}\right)\right)$	(4)
		Explore/exploit ratio (R_EE_)	$R_{\mathrm{EE}}=\frac{saccade\;count\;+\;fixation\;count}{long\;fixation\;count}$	(5)
Dwell	Eye movements comprising a series of fixation-saccade-fixation movements, usually with reference to (or within) a given area of interest.	Dwell count	$d_{n}:\;\;\;\left(x,y\right)\in \left(\left[x_{Min},x_{Max}\right],\left[y_{Min},y_{Max}\right]\right)$ $\forall \;t\in \left[t_{i},t_{j}\right]\;with\;t_{j}\geq t_{i}+30\;ms$	(6)
		Dispersion [17]	$D=\sqrt{{\left(x_{max}-x_{min}\right)}^{2}+{\left(y_{max}-y_{min}\right)}^{2}}$	(7)
Transition	The change of dwell from one area of interest to another and is usually represented in the form of a matrix.	One-/two-way transition probability Transition frequency	e.g., $TM_{OW}=\begin{array}{cc} ROI & \begin{array}{ccc} 1\;\;\;\;\; & 2 & \;\;\;\;3 \end{array}\\ \begin{array}{c} 1\\ 2\\ 3 \end{array} & \left[\begin{array}{ccc} - & p_{1,2} & p_{1,3}\\ p_{2,1} & - & p_{2,3}\\ p_{3,1} & p_{3,2} & - \end{array}\right] \end{array}$	(8)
Scan path	The series of eye movements in accomplishing a specified task. A scan path can include elements of fixations, saccades, dwells and transitions.	Visual entropy [10]	$H=\;-\sum_{i=1}^{n}p\left(X_{i}\right)\sum_{j=1}^{m}p\left(Y_{ij}|X_{i}\right)\;\log_{2}p\left(Y_{ij}|X_{i}\right)$	(9)
		Nearest Neighbour Index (NNI) [12]	$\mathrm{NNI}=\frac{{\bar{r}}_{A}}{{\bar{r}}_{E}}$, where ${\bar{r}}_{A}=\frac{\sum^{\;}r}{N}$ ${\bar{r}}_{E}=\frac{K_{D}}{2\sqrt{N/A}}$	(10)
Pupillo-metry	Measures of pupil size and reactivity.	Pupil dilation spectral power	$P_{dil}=\int_{2Hz}^{6Hz}r\left(\lambda \right)\;d\lambda$	(11)
Blink	Measures of partial or full eye closure.	Blink rate (BLR)	$BLR=\;\frac{n_{blinks}}{t_{i+n_{blinks}}-t_{i}}\times 60$	(12)
		Percentage closure [18,19,20,21]	${\%}_{closure}=\frac{\sum^{\;}t_{closure,\;i}}{t_{Total}}$	(13)

**Table 2:** Qualitative relationships between eye activity variables and selected cognitive states.

Variable	Mental Workload	Attention	Fatigue
Fixation	↑	↑	↑
Blink rate	↑	↓	↑
Saccades	↓	↓	-
Pupil diameter	↑	↑	↓
Visual entropy	↓	↑	-
Dwell time	↓	↑	-

**Table 3:** Heart rate variability time-domain variables adapted from [25], Equations (15)–(18) are included.

Parameter (Unit)	Description	Equation	Equation Number
SDRR (ms)	Standard deviation of RR intervals	$SDRR=\sqrt{\frac{1}{n-1}\sum_{i=1}^{n}{\left(RR_{i}-\bar{RR}\right)}^{2}}$	(15)
SDNN (ms)	Standard deviation of NN intervals	$SDNN=\sqrt{\frac{1}{n-1}\sum_{i=1}^{n}{\left(NN_{i}-\bar{NN}\right)}^{2}}$	(16)
pNN50 (%)	Percentage of successive NN intervals that differ by more than 50 ms	$pNN_{50}=\frac{{\mathrm{count}}_{n-1}\left(\left|NN_{i+1}-NN_{i}\right|>50\;\mathrm{ms}\right)}{n-1}$	(17)
RMSSD (ms)	Root mean square of successive RR interval differences	$RMSSD=\sqrt{\frac{1}{n-1}\sum_{i=1}^{n-1}{\left(RR_{i+1}-RR_{i}\right)}^{2}}$	(18)

**Table 4:** Heart rate variability frequency-domain variables adapted from [25]. Equations (19)–(25) are included.

Parameter (Unit)	Description	Equation	Equation Number
ULF power (ms^2^)	Absolute power of the ultra-low-frequency band (≤0.003 Hz)	$ULF=\int_{0hz}^{0.003hz}f\left(\lambda \right)d\lambda$	(19)
VLF power (ms^2^)	Absolute power of the very-low-frequency band (0.003–0.04 Hz)	$VLF=\int_{0.003hz}^{0.04hz}f\left(\lambda \right)d\lambda$	(20)
LF power (ms^2^)	Absolute power of the low-frequency band (0.04–0.15 Hz)	$LF=\int_{0.04hz}^{0.15hz}f\left(\lambda \right)d\lambda$	(21)
LF power (%)	Relative power of the low-frequency band	$LF\%=\;\frac{\int_{0.04hz}^{0.15hz}f\left(\lambda \right)d\lambda }{\int_{0hz}^{0.4hz}f\left(\lambda \right)d\lambda }\times 100$	(22)
HF power (ms^2^)	Absolute power of the high-frequency band (0.15–0.4 Hz)	$HF=\int_{0.15hz}^{0.40hz}f\left(\lambda \right)d\lambda$	(23)
HF power (%)	Relative power of the high-frequency band	$HF\%=\;\frac{\int_{0.15hz}^{0.4hz}f\left(\lambda \right)d\lambda }{\int_{0hz}^{0.4hz}f\left(\lambda \right)d\lambda }\times 100$	(24)
LF/HF (%)	Ratio of LF-to-HF power	$LF/HF=\;\frac{\int_{0.04hz}^{0.15hz}f\left(\lambda \right)d\lambda }{\int_{0.15hz}^{0.4hz}f\left(\lambda \right)d\lambda }\times 100$	(25)

**Table 5:** Fundamental respiratory variables which Equations (29)–(31) are includes.

Variables (Unit)	Description	Equation	Equation Number
BR (1/min)	Number of breaths per minute.	$BR=\;\frac{n_{breaths}}{t_{i+n_{breaths}}-t_{i}}\times 60$	(29)
TV (mL)	Amount of air inspired in one respiratory cycle	$TV_{i}=V_{peak,\;i}-V_{trough,\;i}$	(30)
MV (L/min)	Amount of air inhaled within one minute	$MV=\mathrm{BR}\;\times \;\bar{\mathrm{TV}}$	(31)

**Table 6:** Qualitative relationships between cardiorespiratory variables and selected cognitive states adapted from [3,33,34,35,36].

Variable	Mental Workload	Attention	Fatigue
HR	↑	↑	↑
SDNN	↓	↑	↑
SDRR	↓	↑	↑
RMSSD	↑	↑	↓
pNN50	↓	-	↓
LF	↑	-	-
HF	↓	-	-
LF/HF	↑	-	↓
Poincare axes	↓	-	-
BR	↓	↓	↓
TV	-	-	↓
MV	-	-	↓

**Table 7:** Comparison of temporal and spatial specifications on electrical and neuroimaging monitoring methods [3].

Category	Electrical Response	Hemodynamic Response
Temporal resolution	High (limited by sampling frequency) [42,43]	Limited (limited by sampling frequency) [44,45]
Temporal sensitivity	High (limited by sampling frequency) [42,43]	Limited (limited by the hemodynamic response of the brain) [46,47]
Spatial sensitivity	Limited (depends on no. of electrodes) [42,48]	High (fNIRS) [45]
Sensitive to movement	Sensitive to eye, head, body and etc. movement. Noise filtering algorithms are required.	Might need to filter out heart activity from the raw measurements.
Intrusiveness	More intrusive [42]	Low

**Table 8:** Summary of neuroimaging techniques as indicators of cognitive states [3].

	Mental Workload	Engagement/Attention/ Vigilance	Working Memory	Fatigue
EEG	Spectral ratio [51,52,53] Spectral bands [54,55,56,57,58,59,60] Regression [61] Bayesian modelling [53] Neural networks [62,63,64,65,66,67] Multivariate analysis [68,69,70] Discriminant analysis [66,71,72,73,74,75,76]	Spectral ratio [77,78,79] Spectral bands [52,56,80,81] Committee machines [82,83,84] Discriminant analysis [75,85]	-	Multivariate analysis [69] Discriminant analysis [75]
fNIRS	oxy-hemoglobin (HbO), deoxy-hemoglobin (HbR) [86,87,88,89,90,91,92,93,94]	Oxygenation wave size [91,95,96]	HbO, HbR [97,98,99]	HbO, HbR [100]

**Table 9:** Summary of validity of BioHarness in heart rate measurement.

Subject	Physical Testing		Mental Testing	
	RMS Error	*CC*	RMS Error	*CC*
1	0.0953	0.9153	0.0345	0.7878
2	0.0276	0.8839	0.0148	0.8997
3	0.1386	0.6312	0.1113	0.7008

**Table 10:** Cluster centres for heart rate and breathing rate for mental workload in ATM scenario.

	HR (L/min)	BR (L/min)
Low	63.2	11.5
Medium	64.9	14.6
High	68.3	15.3
